# Market liquidity and volatility: Does economic policy uncertainty matter? Evidence from Asian emerging economies

**DOI:** 10.1371/journal.pone.0301597

**Published:** 2024-06-11

**Authors:** Zumara Muzaffar, Imran Riaz Malik

**Affiliations:** Management Sciences, Capital University of Science and Technology, Islamabad, Islamabad Capital Territory, Pakistan; Universiti Malaysia Sabah, MALAYSIA

## Abstract

This research investigates the complex interaction between liquidity and volatility while considering Economic Policy Uncertainty (EPU) as a moderating factor. Using a comprehensive dataset that incorporates various liquidity measures such as market resilience, depth, and breadth, the study examines how changes in liquidity impact volatility in four Asian incipient economies: China, Pakistan, India, and South Korea. By utilizing sophisticated econometric techniques, particularly the System Generalized Method of Moment (GMM), the findings demonstrate a statistically significant inverse relationship between liquidity and volatility. These findings imply that, within the Asian context, lower levels of volatility are correlated with higher market liquidity. By incorporating EPU into the model, the research acknowledges the significant role of economic factors in shaping market dynamics. Stakeholders, decision-makers, and investors can gain valuable insights from this analysis of variables influencing market stability in Asian emerging economies. The study’s outcomes can guide policymakers in formulating strategies that promote market stability and improve market microstructure.

## Section 1: Introduction

The world economy has expanded in recent years, and it has displayed noticeable patterns of periodicity and volatility. A specific aspect prone to stock price sensitivity is associated with abrupt changes that are frequently linked to liquidity problems [1, 2]. The key method for distributing financial resources is liquidity, which is regarded as the stock market’s lifeline. Current literature emphasizes, the importance of liquidity and volatility, which are considered the two most important aspects of financial markets, considering the close association and shared characteristics. Liquidity is defined as the ease of buying or selling any asset, stock, or bond. It also refers to the ability to execute a large trade quickly at the minimum cost and with a low price impact [3]. On the other hand, volatility is linked to uncertainty and illustrates the extent to which asset prices fluctuate [4]. Stakeholders, corporations, and regulatory agencies must maintain a consistent level of liquidity in the stock market. This consistency ensures consistent trading at target prices and regulates the cost of acquiring capital [5, 6].

The extant literature provides mixed evidence on the relationship between liquidity and volatility. For instance, a few studies have explored the relationship and shown positive relationships. [7] Suggests that volatility reflects liquidity, particularly in deep markets, which appear less volatile than thin markets [2] Establish a correlation between liquidity and volatility, linking global financial crises (2008–2009) to liquidity crunches that intensified volatility [8]. Find a robust relationship between liquidity and volatility, with variations across countries: emerging markets generally exhibit lower liquidity and higher volatility compared to developed countries. Drawing from the market microstructure approach, [9] presents an information-based model predicting negative relationships between liquidity and volatility. In this model, informed traders possess insider knowledge, impacting asset supply and demand by widening bid-ask gaps. The widening bid-ask spread increases transaction costs, this whole activity reduces liquidity and instigates volatility in the stock market. Conversely [10] argues for a positive relationship between volatility and liquidity [11] proposes an aggressive relationship between market liquidity and volatility, emphasizing that even a small change in volatility leads to a substantial change in market liquidity. The global financial and economic crisis erupted in 2008, leaving the quest for a complete understanding of the mechanics of stock market volatility and liquidity as the distinguishing intense priorities confronting stock market investors and regulators globally [12]. There are various hypotheses and schools of thinking about the relationship between stock market liquidity and volatility at the index level. The existing literature predominantly focuses on the correlation between these variables and the impact of volatility on liquidity, with limited research on how liquidity influences volatility. Addressing this gap requires empirical investigation, especially in the context of Asian emerging economies.

The recent coronavirus (COVID-19) pandemic has introduced an unprecedented level of uncertainty worldwide, affecting not only our daily lives and public health systems but also significantly impacting the global stock markets. Alongside this pandemic, due to the onset of the global financial crisis in 2007, the European debt crisis, and political uncertainty, have garnered significant scholarly interest during the past ten years. Early indications of uncertainty literature can be found in the writings of [13–17], Created the Economic Policy Uncertainty Index (EPU) based on the frequency of media coverage to evaluate the impact of policy uncertainty. There are abundant empirical pieces of evidence that claim financial markets are also responsive to changes in aggregate uncertainty such as [18–21]. In addition, in recent years ample studies have emerged that observed innumerable ways through which policy uncertainty affects financial variables. [22–27].

At the firm level, changes in economic policies affect firm performance, thereby increasing operational risks. However, at the stock market level investors, faced with uncertainty about future market directions and policy developments, may withdraw funds from the market to mitigate investment risks, influencing the efficacy of policy interventions on the overall economy [28]. This uncertainty extends to capital markets, prompting investors to delay or halt business and investment activities, resulting in an abrupt reduction in stock market liquidity [29, 30]. The relationship between market liquidity and economic policy uncertainty is being examined by [31], noting intensified co-movements during crises and the contribution of illiquidity to EPU. Moreover, financial economists are keenly interested in understanding the EPU phenomenon from the asset-pricing perspective as it reduces returns and induces volatility in financial markets [32–34]. Empirical investigations into the relationship between EPU and stock market return and volatility have been a significant focus [35, 36]. In this regard, [37] thoroughly examined the impact of economic policy uncertainty on return and volatility under different market conditions, including bearish and bullish trends in G-7 countries. They discovered that an increase in EPU leads to heightened volatility, with this trend being more pronounced in bearish markets (less deep/thin) compared to bullish markets (thicker/deeper), resulting in more substantial stock price declines in bear markets due to increased uncertainty.

The extensive body of research underscores the influence of EPU on volatility and liquidity. Examining EPU as a factor interacting with volatility and liquidity becomes imperative because economic uncertainties directly or indirectly affect the efficiency and performance of the stock market. Consequently, there is a need for empirical investigations to understand how economic uncertainty shapes the causal relationships among financial variables at the microstructure level. This presents an avenue for further research into the intricate links and moderating effects of economic policy uncertainty on the effective relationship between liquidity and volatility in financial markets.

The Efficient Market Hypothesis (EMH) suggests that stock prices accurately incorporate all available information, including economic conditions and policy announcements. Fama (1970) categorized empirical efficient markets into three forms: weak form, semi-strong form, and strong form. The weak form includes historical information, the semi-strong form encompasses historical prices and publicly available information, including announcements related to monetary policy. This study focuses on the relationship between liquidity and volatility, aiming to reveal how efficiently markets process and respond to the incorporation of new information. Sudden changes in Economic Policy Uncertainty (EPU) are examined in connection with variations in liquidity and volatility, which may indicate that market participants are reacting to and assimilating new information. The Adaptive Market Hypothesis (AMH) extends the EMH by emphasizing the evolutionary nature of markets and the adaptive behavior of participants. If the relationship between liquidity, volatility, and EPU reflects adaptive responses to changing conditions, it aligns with the principles of the AMH.

This study makes the following contributions. In comparison, the bulk of past research focused on single-security level or single-economy-based analysis of volatility and liquidity [6]. This study took into consideration the stock Index markets of four Asian emerging economies i.e. China, Pakistan, India, and South Korea, and a data set comprised of monthly data from year 2004 to 2020. Secondly, this study also intrudes regulatory economic factor EPU as a moderator as [38, 39], incorporated in their studies while previous studies were confined to the direct relation of regulatory economic variables with liquidity and volatility [40, 41]. The third contribution pertains to the methodological framework, the two-step System GMM estimator has been adopted which is robust in that, it produces consistent and dependable estimates, [10] applying the same techniques for estimating the relationship between liquidity and volatility. The fourth contribution is the liquidity index has been developed, so the perspective of the multidimensional aspect is also covered in this paper as [42] conducted their studies on liquidity and volatility proxies.

The remainder of this paper is structured as follows: Section 2 provides an overview of the existing literature concerning the connections between liquidity and volatility, liquidity, and Economic Policy Uncertainty (EPU), as well as volatility and EPU. Section 3 outlines the variables considered, the estimation methodology employed, and the regression analysis conducted to explore the relationships between liquidity and volatility, incorporating EPU as a moderator. Section 4 explores the results obtained and highlights the contributions made to the existing literature. Section 5 is specifically focused on the conclusion of the study. Lastly, Section 6 presents recommendations based on the findings and insights derived from the research.

## Section 2: Literature review

Liquidity holds a crucial role in both risk measurement and asset pricing [43]. The impact of liquidity on volatility has been extensively explored in the literature [44, 45]. Previous studies generally suggest that higher liquidity tends to decrease volatility, indicating a negative relationship between liquidity and volatility [46]. Empirical research by [4] also points towards an inverse link between volatility and liquidity, potentially attributed to the presence of informed traders [8]. Identify a robust correlation between volatility and liquidity, emphasizing that variations exist on a national level. [2] Argue that market volatility and liquidity dynamics are interconnected through their theoretical model [42]. Investigated that liquidity proxies and volatility proxies reveal a causal association between high and low liquidity ratios and volatility [47] investigated the relationship between various liquidity dimensions and asset price volatility in China, finding that while liquidity can explain high or low volatility in a stock market, it cannot explain volatility concerning the expansion or contraction of liquidity [48]. Studied the causal relationship between different liquidity proxies and volatility, revealing that liquidity proxies (VOV, HLR, LTV) have a causal relationship with risk proxies (VORET, SQRET) [49]. focused on the efficiency of the crypto-currency market, indicating that higher liquidity improves crypto-currency efficiency, while higher volatility weakens it [50]. Empirically analyzes the relationship between stock market turnover and stock volatility in the Nigerian stock market from year 1997 to 2019 and reveals that liquidity has a negative relation with volatility [6]. analyzed the impact of liquidity on volatility in the Zimbabwe stock market, they used bid-ask spread and trading volume as proxies of liquidity. They applied the system GMM model for estimating the empirical relationship and revealed that trading volume has a significant positive impact, while bid-ask spread has a negative relation with volatility. They found that high liquidity with low volatility facilitates arbitrage opportunities and enhances market efficiency.

Empirical research on the connection between political unpredictability and asset pricing is growing in popularity [51]. Their theoretical models [52, 53], also claim when uncertainty increases, the trading volume of one’s stock market drops, as arbitrageurs halt trading, widening the bid-ask spread that causes great fluctuation in asset price [46, 54], Use the policy uncertainty index developed by [17] who pointed out that the introduction of a new policy whose influence is uncertain or unpredictable might increase the volatility. Time-varying relationships between policy uncertainty, implied volatility, and U.S. stock market returns are found by [35], who also find that a rise in EPU causes an increase in stock market volatility.

However, [41] investigates the relationship between the uncertainty of economic policy and the liquidity of the Brazilian stock market. Particularly for companies with higher risk profiles in a developing order-driven stock market, there is a discernible increase in stock illiquidity during periods of heightened economic policy uncertainty. According to [40], economic policy uncertainty affects liquidity moderately under normal conditions but becomes quite important when there is a major financial crisis.

### Hypotheses development

Numerous factors influence the stock-market volatility. These include, but are not limited to, liquidity, macroeconomic factors, stock returns, etc. However, liquidity and volatility are immensely studied variables in the field of finance, especially in the field of market microstructure, since they are of much importance to policymakers, investors, and regulators. Speculators, hedgers, and arbitrageurs are among traders who adopt different investment strategies. Their investment strategies are based on the liquidity position of the stock market and aspects of asset pricing i.e. return and volatility. In addition, the traders even alter their investment strategy in response to fresh information that enters the market in an attempt to forecast potential future investment prices. According to the policymaker and regulators’ view, the hindrance in market activity is caused by these fundamental variables i.e. Liquidity and volatility [10] As a result, the study of liquidity and volatility is relevant in the market microstructure literature. A recent study conducted by [6, 47] on this relationship by applying GMM posited a negative association between liquidity and volatility. Therefore, the following hypothesis is being postulated based on the results of the above literature.

H_1_: Market liquidity has a negative relation with the volatility of the stock market.

The above literature empirically and theoretically illustrates that any economic uncertain news significantly impacts financial variables i.e. liquidity and volatility, especially in those stock markets that are less deep and less resilient such as [38, 52, 53, 55]. Furthermore, [39] uses panel data of 457 listed banks in 20 countries to empirically investigate the impact of bank liquidity on volatility with the moderating effect of EPU in the banking sector by taking a panel dataset of 457 banks of 20 countries. Empirical analysis illustrates bank liquidity hoarding has a significant negative contribution towards volatility. In addition, EPU as a moderator further strengthens the negative relation between liquidity and volatility. Through lab tests, [38] investigate how liquidity affects volatility. They find that in markets with less liquidity, traders are highly sensitive to uncertain news related to asset values. Uncertain news triggers a liquidation process, leading to a liquidity crunch and causing fluctuation in asset pricing. Moreover, [6] also ascertains that according to the policymakers’ and regulators’ myopic view, the uncertain economic news is a possible cause of hinges on the market activity. Considering that economies are less deep, less resilient, and have low trading activity, an increase in EPU may cause fluctuation in asset pricing. The EPU as a regulatory economic variable is likely to affect the relationship between liquidity and volatility. Therefore, the following hypothesis is being postulated based on the results of the above literature.

H_2_: EPU as a moderator strengthens the negative relationship between market liquidity and volatility of the stock market.

## Section 3: Methodology

In this study, a panel data research design is employed, utilizing monthly data from the years 2014 through 2020. The data on Economic Policy Uncertainty (EPU) is sourced from www.policyuncertainty.com, while volume and stock price data are obtained from https://www.investing.com/indices/world-indices. The focus of this study will be on Asian emerging countries. South Korea, China, India, and Pakistan. These economies are in a rapid growth phase, attracting foreign and institutional investors despite facing instability and volatility issues. To create a more attractive investment environment, it is crucial to comprehend the dynamics of the stock market, mitigate economic uncertainties that trigger volatility, and enhance liquidity provision at the micro level in these Asian emerging economies. To assess overall liquidity, the study creates a liquidity index, incorporating three measures of liquidity: market resilience, market breadth, and market depth [48]. An approach is adopted to formulate an index that considers three key liquidity factors: tightness, resilience, and market depth.

The problem of endogeneity in panel data is controlled by applying dynamic panel analysis, also called the generalized method of moments (GMM). The generalized method of moments is a methodology that addresses endogeneity by taking the lagged dependent variable and endogenous factors with appropriate lags as instrumental variables. Therefore, Generalized Methods of arise because of the simultaneity or bias of omitted variables [56]. The generalized method of moments has two types, one is called difference GMM, and another one is system GMM. In difference GMM, the differenced equations are taken only, while in system GMM both difference and level equations are considered. In this study, the system GMM estimator would address issues related to serial correlation, hetero-scedasticity, and endogeneity of variables [57–59]. The use of system GMM facilitates robust analysis and enhances the ability to handle complex dynamics in the panel data [60]. Furthermore, two post-estimation tests will be applied to test the validity of the econometric model and to verify whether instruments are correctly specified or not, these tests are (1) the Sargan test; and (2) the Arello-Bond test for first-order correlation [60].

### Estimation equation


$$\nu_{it}={ß}_{0}+{ß}_{1}{ΜLΙ}_{it}+{ß}_{2}{EPU}_{it}+{ß}_{3}{\left(MLI)(EPU\right)}_{it}+\varepsilon_{it}$$ (1)

In this equation, we focus on the volatility *v*_*it*_ as the dependent variable in the year t. The Market Liquidity Index MLI_*it*_ at year t, drawn from a sample size of n, and Economic Policy Uncertainty EPU_*it*_ at year t for the same sample size of n, are crucial factors. The interaction term MLI*EPU_*it*_ effect at year t in the sample size n. The error term is denoted as Ɛₜ, capturing unobserved factors. The coefficients β₀, β₁, β₂, and β₃ represent the parameters to be estimated through the regression equations, outlining the relationships within the model.

### Variables

According to [9, 61], market liquidity is a complicated and perplexing concept that cannot be studied using a single dimension. [9] Defines three important dimensions of market liquidity: market depth, tightness, and resilience [62]. Added two new factors to this paradigm, namely immediacy and market depth. Liquidity will be examined in three dimensions in this study: market depth, market breadth, and market resilience. The choice of these dimensions is influenced by their low-frequency measure, which is generated from volume data and indicates strong liquidity Le and [63, 64]. The definitions of the selected dimensions are as follows.

Market Resilience: Market resilience is defined as the rate or speed at which prices can rebound after a random, uninformative shock [9]. These uninformative shocks are frequently caused by high trading volume or stock-related news. The market is deemed to be resilient when prices quickly revert to fair levels. The market resilience will be measured by the conventional liquidity ratio [65]. The formula of the conventional liquidity ratio is. *MR* Represents market resilience, *P*_t_ stands for today’s price, *V*_t_ stands for volume, and *P*_*t*−1_ stands for yesterday’s price.


$$MR=\frac{P_{t}V_{t}}{\left|P_{t}-P_{t-1}\right|}$$ (2)

Market depth: Market Depth, as defined by [62], refers to the stock market’s ability to survive against relatively large market orders without causing significant price impacts on the asset. The market turnover ratio will be used to assess market depth.

Market breadth: The market breadth defined by [9] describes the bullish and bearish trend of the stock market by analyzing the comparative change in the advance movement of stocks to declining stocks. The market breadth will be gauged by the Amivest liquidity ratio. The formula of the Amivest liquidity ratio is. MB represents market breadth, V_t_ denotes volume at time t, and l rt l represents absolute return.


$$MB=V_{t}/l\;r_{t}\;l$$ (3)

Volatility: In this paper, volatility is measured by taking the log difference between high and low prices. It is proposed by [4, 66]. The formula for measuring volatility is as follows.


V=ln(H)−ln(L) (4)

Economic Policy Uncertainty: In this study, EPU will be assessed by using the EPU Index proposed by [17]. The EPU Index is considered a standard measure and has been extensively used in finance and economics literature [31, 40, 67]. It is a comparatively superior metric than the ones because, among other things, it captures the time-varying uncertainties in the environment resulting from many sources of economic policy concerns [30, 68]. The EPU index is being developed using articles from reputable newspapers that are especially about policy uncertainty [17], it totals the number of newspaper articles that contain the terms unsure or uncertain, economic, or economy, and one or more terms related to policy, such as "legislation," "congress," "deficit," and "Federal Reserve."

## Section 4: Discussion and empirical analysis

Table 1 presents the descriptive statistics of panel data and individual countries. The period of the study spreads from 2004 to 2020 with 860 monthly observations. The range of monthly data of volatility is from 1% to 54%. Volatility has an average value of 9% with a total deviation of 6%. The reason for the high volatility is due to the financial crises of 2008–2009 and the pandemic coronavirus. The range of monthly data of the Liquidity index is 3.48% to 4.59% and the average mean is 3.91% to a standard deviation of 0.36. This reflects that the liquidity of selected Asian economy’s stock markets behave normally, not seeing bullish and bearish trends. The EPU standard deviation is 0.55 with a mean value of 4.64%, a range of dispersion of 3.16% to 6.49%, Compared to volatility EPU shows less dispersion. The skew-ness values of each variable of panel data lie between 0.66 to 2.68. This shows that the data is moderately skewed. The kurtosis value lies between 2.62 to 13.88 and shows that data distribution is leptokurtic distribution.

**Table 1:** Descriptive statistics of panel data.

**Variables**	**Mean**	**Std.Dev**	**Maximum**	**Minimum Kurtosis Skew-ness**
**Volatility**	0.09	0.06	0.01	0.01 2.68 13.88
**Liquidity Index**	3.91	0.36	3.48	3.48 0.83 2.19
**EPU**	4.64	0.55	3.16	3.16 0.25 3.22
Liquidity Index*EPU	18.35	3.85	29.81	29.81 0.66 2.62
***Descriptive Statistics of China Data***				
**Variables**	**Mean**	**Std.Dev**	**Maximum**	**Minimum Kurtosis Skew-ness**
**Volatility**	0.16	0.07	0.54	0.10 2.22 9.12
**Liquidity Index**	4.50	0.12	4.59	3.96 3.67 14.90
**EPU**	5.34	0.34	6.49	4.95 1.08 3.59
Liquidity Index*EPU	24.07	1.88	29.81	0.12 0.25 3.70
***Descriptive Statistics of India Data***				
**Variables**	**Mean**	**Std.Dev**	**Maximum**	**Minimum Kurtosis Skew-ness**
**Volatility**	0.08	0.008	0.10	0.07 0.17 1.87
**Liquidity Index**	3.88	0.03	3.96	3.83 0.03 2.28
**EPU**	4.79	0.09	4.95	4.64 -0.06 1.87
Liquidity Index*EPU	18.64	0.50	19.63	17.78 -0.02 1.97
***Descriptive Statistics of Pakistan Data***				
**Variables**	**Mean**	**Std.Dev.**	**Maximum**	**Minimum Kurtosis Skew-ness**
**Volatility**	0.06	0.006	0.07	0.05 -0.09 1.81
**Liquidity Index**	3.70	0.07	3.83	3.59 -0.20 1.52
**EPU**	4.47	0.11	4.64	4.24 -0.19 1.81
Liquidity Index*EPU	16.59	0.76	17.78	15.31 -0.16 1.66
***Descriptive Statistics of South Korean Data***				
**Variables**	**Mean**	**Std.Dev**	**Maximum**	**Minimum Kurtosis Skew-ness**
**Volatility**	0.04	0.008	0.05	0.01 -0.78 3.10
**Liquidity Index**	3.56	0.02	3.59	3.48 -0.65 3.03
**EPU**	3.95	0.23	4.26	3.26 -0.92 3.35
Liquidity Index*EPU	14.08	0.93	15.29	11.01 -0.87 3.24

This table presents the descriptive statistics of the following variables—Volatility, Liquidity Index, EPU (Economic Policy Uncertainty) of panel data and individual countries i.e. China, India, Pakistan, and South Korea. The descriptive statistics include mean, maximum, minimum, skew-ness, and kurtosis values.

The descriptive data of individual countries in Table 1 shows that China’s mean value of volatility is 16.9% with a standard deviation of 0.07. That shows that financial debt crises and COVID-19 hit China the most as compared to the other three countries. India, Pakistan, and South -Korea’s average value of volatility is 8%, 6%, and 4% with a standard deviation range from 0.006 to 0.008. However, the dispersion of volatility is extremely high in India, Pakistan, and South Korea as compared to China. This indicates a remarkable oscillation in stock prices in the focused period in all four countries. The Liquidity factor in all four stock markets behaves normally with a minimum of 3.48% to a maximum of 5.40% the average value of liquidity in China is 4.50% while in other countries average liquidity is 3.80%, 3.70%, and 3.69%. The liquidity and volatility data shows though financial and pandemic crises hit China at the extreme, it has enormous reserves that mitigate the risk factor to some extent. Similarly, the average value of the EPU of China is 5.34% with a standard deviation of 0.34. The other countries’ average values are 4.47%, 4.49%, and 3.58%. That reflects that economic uncertainty prevails high in China than other three countries. The skew-ness value lies between the ranges of 0.25 to 3.67 reflecting that data is highly skewed. The skew-ness value of India data lies between -0.02 to 0.17 reflecting that data is symmetrical. The skew-ness value of Pakistan data lies between -0.2 to -0.09 which reflects that data is moderately skewed. The skew-ness value of South Korea data lies between -0.92 to -0.87 reflecting that data is moderately skewed. The kurtosis value of China data is greater than 0, depicting that data is distributed heavily on the left tail and its leptokurtic distribution. However, the kurtosis values of Pakistan, South Korea, and India lie near 3.8 to 1.87 which shows data distribution is not so much heavy-tailed but still falls in the category of leptokurtic distribution.

The results showcased in regression Table 2 provide valuable information into the connection between the Liquidity Index, volatility, and the moderating impact of Economic Policy Uncertainty (EPU). Notably, the Liquidity Index demonstrates a compelling significance with a P value of 0.000 at a 5% confidence interval. The corresponding coefficient of -14.41% signals a significant and negative association this proves H1, on the panel data of Asian emerging economies. The results of the H_1_ are in favor of the findings of [4, 42, 47–50]. They all observed negative relationship between liquidity and volatility can be attributed to fund providers restricting their involvement in the stock market when facing bearish trends. This cautious approach prompts the withdrawal of funds through share sales. Consequently, there is a collective effect of reduced liquidity availability, which is closely tied to an escalation in volatility. Furthermore, the analysis indicates that in emerging economies with less robust markets and lower resilience to market volatility, the ease with which buyers and sellers can adjust prices contributes to diminished liquidity. This, in turn, exacerbates fluctuations in asset pricing. However, [6] applied the system GMM to estimate the liquidity and volatility association.

**Table 2:** The system GMM model.

Variables *β*_0_	*β*_1_	*β*_2_	*β*_3_	Sargan Test A1
P-values 0.000**	0.000**	0.000**	0.000**	0.1804 0.000
**Coefficients 0.384**	(0.1441)	(0.0414)	0.102	

This table presents the regression analysis which includes coefficients, p-value at a 10%,5%, and 1% significance level, and the Sargan Value is insignificant i.e. 0.1804. The insignificant value shows non-rejection of the null hypothesis that endogeneity is present. A1 presents auto-correlation as significant at level 1.

As, [17] and many other scholars have established economic policy uncertainty (EPU) has been of great significance to financial activities [16]. It is expected that EPU may play an important role in the relationship between liquidity and volatility, as volatility is closely related to financial activities. Therefore, by testing Eq (1) to assess how EPU affects the relationship between liquidity and volatility in Asian Emerging economies. The results showcased in regression Table 2 provide valuable information into the connection between liquidity, volatility, and the moderating impact of Economic Policy Uncertainty (EPU). The interaction term (Liquidity*EPU), boasting a P value of 0.000 and a coefficient value of 10.2%, reveals a substantial positive relationship and acceptance of hypothesis *H*_*2*_. This positive interaction term amplifies the previously identified negative association between liquidity and volatility. There is clear evidence that EPU in Asian economies enhances the negative relationship between liquidity and volatility. This suggests that in thin markets, during periods characterized by heightened uncertainty, often triggered by sudden shifts in economic policies, the stock market tends to undergo decreased liquidity and subsequent increased volatility as high volatility is associated with high EPU during a bearish trend [69]. Admittedly, the EPU being a moderator significantly affects the relationship of financial variables and hinges on market activity [6]. The empirical results of this study are consistent with the previous work of [37–39]. This study also gives insight into the influential power of EPU on the performance of the Asian emerging stock. So it is indeed important that policymakers of these economies devise investment strategies that understand the dynamics of EPU and formulate such risk-management techniques to mitigate its shock, for stabilization of the stock market. The post-diagnostic test for robustness and validity, particularly the Hansen-J over-identification test, generates a test statistic of 1.79 with a probability value of 0.1804. The non-rejection of the null hypothesis, which asserts the absence of endogeneity and overly restrictive identification, provides a boost to confidence in the reliability and validity of the instruments utilized in the analysis. This implies that the instruments employed in the study are robust and appropriately identified, strengthening the trustworthiness of the analytical results. A1 is significant as a p-value of 0.0001, which presents there is no autocorrelation at level 1.

This study made a major contribution in the areas of asset pricing, risk management, and marker-microstructure and to the literature such as [6, 47, 50]. They applied the system GMM technique to estimate a negative association between liquidity and volatility. Moreover, it also sheds light on the information-based- model of [9]. The empirical findings of this study contrast with [49, 70] all stated a positive connection between volatility and liquidity. Further, this study provides empirical evidence that Asian emerging stock markets, under the condition of EPU, become more volatile followed by low liquidity, and supports the theoretical [52, 53] and empirical findings [38, 39]. This further proves that Asian emerging economies are not well efficient in assimilating such information into prices and financial and economic shocks play a vital role in affecting stock market efficiency. Financial variables are also responsive to changes in aggregate uncertainty. So this study also contributes to AMH (Adaptive market hypothesis).

## Section 5: Conclusion

This study investigates the interaction between the market liquidity index and volatility within the context of four emerging economies in Asia. The pragmatic results indicate a notable negative impact of the market liquidity index on volatility, possibly attributed to the asymmetric information distribution among different traders, as per the market microstructure approach. Furthermore, the incorporation of various liquidity characteristics into the index enhances its utility and adaptability across diverse market conditions, offering a more comprehensive understanding of liquidity dynamics. The study extends its focus to examine the interaction between the market liquidity index under the discussion of Economic Policy Uncertainty (EPU). Empirical findings indicate that market liquidity has a more pronounced impact on volatility during periods characterized by elevated economic uncertainty. These results underscore the intricate dynamics within financial markets, emphasizing a discernible negative relationship between market liquidity and volatility, which is further influenced by the moderating effect of EPU. Several factors contribute to the observed negative association. Economic policy uncertainty introduces ambiguity, heightening investor apprehension and hesitation, thereby diminishing market liquidity. Moreover, uncertainty arising from economic policy decisions can magnify market reactions, resulting in increased volatility. Recognizing and understanding these dynamics are pivotal for making well-informed decisions in financial markets. The insights from the study provide valuable considerations for policymakers and market participants navigating these intricate relationships, offering a foundation for strategic decision-making in the face of economic policy uncertainties. The inclusion of market resilience, market depth, and market breadth into a comprehensive liquidity index provides a nuanced understanding of liquidity circumstances in financial markets. This information is pertinent for researchers, policymakers, investors, and risk managers seeking to make informed decisions and manage risks effectively.

## Section 6: Recommendations

In financial markets, the relationship between liquidity and volatility is intricate, necessitating a comprehensive understanding for policymakers and market participants. Recognizing how liquidity influences volatility is crucial for informed decision-making and effective market functioning. Policymakers play a pivotal role in fostering market transparency. Providing investors with more information regarding liquidity conditions in the stock market can enhance overall transparency. Increased transparency helps reduce information asymmetry among market participants, enabling them to make more informed and prudent trading decisions. Regular assessments and reviews of regulatory frameworks and policies are essential to ensure they promote sound market liquidity. Policymakers should actively engage in ongoing discussions to gain insights into emerging risks and to ensure that market participants are well-informed about regulatory requirements. This dynamic approach allows for adaptability to changing market conditions and helps maintain a resilient and efficient financial system. In summary, policymakers should focus on promoting transparency, regularly reassessing regulatory frameworks, and engaging in ongoing discussions to ensure that market conditions remain conducive to sound liquidity and informed decision-making by market participants.

### Future directions

Future research could extend to other asset classes such as (bonds, commodities, and equities) and other economies like frontier economies. Future research could be done on more dynamic models such as the vector auto-regression (VAR) model or dynamic conditional correlation (DCC) model, to subtle the changing nature of these variables over the period. Nowadays, Environmental, Social, and Governance (ESG) is an evolving and new factor in investment decision-making. In the future researchers could investigate how ESG interacts with liquidity and volatility dynamics.
